# The gut microbiota diversity of five Orthoptera (Insecta, Polyneoptera) insects determined by DNA metabarcoding

**DOI:** 10.3897/BDJ.11.e98162

**Published:** 2023-03-15

**Authors:** Yantong Liu, Lina Zhao, Zhongying Qiu, Hao Yuan

**Affiliations:** 1 School of Basic Medical Sciences, Xi’an Medical University, xi'an, China School of Basic Medical Sciences, Xi’an Medical University xi'an China; 2 College of Life Sciences, Shaanxi Normal University, xi'an, China College of Life Sciences, Shaanxi Normal University xi'an China

**Keywords:** gut microbiota, DNA metabarcoding, Orthoptera, biodiversity

## Abstract

Most orthopteran insects are phytophagous and some are important pests in agriculture and forests. Many intestinal microflora of Orthoptera insects have been reported, primarily from Acridoidea and there have been few reports of other taxa. In this study, we collected 15 individuals representing five species (*Ruspolialineosa*, *Tetrixjaponica*, *Erianthusversicolor*, *Gryllotalpaorientalis* and *Teleogryllusemma*) belonging to five orthopteran superfamilies (Tettigonioidea, Tetrigoidea, Eumastacoidea, Gryllotalpoidea and Grylloidea) to characterise and compare the gut microbiota with greater taxonomic width by performing sequencing analysis of the 16S rRNA V4 region in gut material. A total of 606,053 high-quality sequences and 3,105 OTUs were acquired from 15 gut samples representing 24 phyla, 48 classes, 69 orders, 133 families and 219 genera. Firmicutes and bacteria were the most abundant phyla, followed by Bacteroidetes, Cyanobacteria, Actinobacteria and Acidobacteria. At the genus level, *Serratia*, *Citrobacter*, *Wolbachia*, *Lactobacillus* and *Parabacteroides* were the most predominant genera in *R.lineosa*, *T.japonica*, *E.versicolor*, *G.orientalis* and *T.emma*, respectively. Both Principal Coordinates Analysis (PCoA) and heatmap results revealed significant differences in bacterial community composition across species. Additionally, alpha diversity analysis indicated the bacterial richness was significantly different amongst the five species.

## Introduction

Large numbers of microorganisms colonise the insect gut and form complex symbiotic relationships with their host. Insect-gut symbiotic microorganisms play important roles in parasitifer mating preference (Sharon et al. 2010), resistance to harmful microbes (Scott et al. 2008), expand the range of diet (Anonymous 2008), longevity (Behar et al. 2008), the regulation of phenolic compound bioavailability (Selma et al. 2009) and pheromone aggregation (Dillon and Charnley 2002). In addition, symbiotic microorganisms in the insect gut influence parasitifer nutrition, digestion and the immune response. Recent work has indicated that insect symbionts mediate insecticide resistance. Studies investigating the mid-gut microbiota of the diamondback moth have suggested roles for Lactobacillales or other scarcer taxa in conferring diamondback moth insecticide resistance (Xia et al. 2013). Many factors influence insect gut communities. Changes in the gut ecological conditions impact the structure and diversity of bacterial populations; for example, variations in the physicochemical conditions in different gut compartments of *Cubitermes* spp. are reflected in the diversity of their respective intestinal microbial communities (Schmitt-Wagner et al. 2003). Furthermore, sampling site location primarily reflects microbiota composition rather than taxonomy or ecology (Hird et al. 2014). According to a recent report, gut bacterial diversity is significantly higher in omnivorous insects than in stenophagous insects (Yun et al. 2014) and higher bacterial diversity may be related to the types of food consumed (Anderson et al. 2013). Dillon and Charnley studied the numbers and types of intestinal microflora in *Schistocercagregaria* and demonstrated how different diets influenced gut microbe numbers and varieties (Dillon and Charnley 2002). Shi et al. studied the microbial community structures of gut symbionts in woodbore, silkworm, grasshopper and cutworm and observed significant differences in symbiotic community structure correlated with food adaptation (Shi et al. 2011). However, because traditional sequencing technology is low-throughput and time-consuming, the exploration of insect gut bacterial diversity has been limited.

DNA metabarcoding, a high-throughput DNA barcoding technique, is a fast and efficient method to assess biodiversity (Yu et al. 2012, Carew et al. 2013, Leray and Knowlton 2015, Dowle et al. 2016). This approach has aroused widespread interest amongst scientists and has been widely employed to investigate soil, water, intestines, air and other ecologies (Chen et al. 2014, Kraaijeveld et al. 2015, Xiong et al. 2015, Zhao et al. 2015, Yu et al. 2015). DNA metabarcoding technologies facilitate accurate, rapid and highly efficient identification on a large scale and, to a large extent, compensate for the defects of traditional identification methods. DNA metabarcoding has been widely employed to study the intestinal microflora of insects. For example, Minard et al. performed DNA metabarcoding sequencing to compare the intestinal microflora of four autochthonous *Aedesalbopictus* populations in Vietnam and three populations recently introduced to metropolitan France and found that French invasive Asian tiger mosquito populations harbour reduced bacterial microbiota and genetic diversity compared to Vietnamese autochthonous relatives (Minard et al. 2015). According to Gauthier et al., who analysed the diversity of bacterial communities associated with nine biotypes of the pea aphid complex via DNA metabarcode sequencing, the aphid microbiota is dominated by a few heritable symbionts and plant specialisation is an important structural factor for bacterial communities associated with the pea aphid complex (Gauthier et al. 2015). The widespread use of DNA metabarcoding technology has revolutionised the study of insect intestinal microflora.

Most orthopterans are phytophagous and some are important pests in agriculture and forests. Most reports of intestinal microflora in Orthoptera have primarily concentrated on Acridoidea (Dillon et al. 2008, Idowu et al. 2009, Ademolu and Idowu 2011) and there have been few reports of other taxa. In this study, we used DNA metabarcoding to investigate the gut microbial composition and diversity in five superfamilies (Tetrigoidea, Eumastacoidea, Tettigonioidea, Gryllotalpoidea and Grylloidea) of Orthoptera.

## Material and methods

### Insect sampling

A total of 15 orthopteran specimens across five species (*Ruspolialineosa* belonging to Tettigonoidea, *Gryllotalpaorientalis* belonging to Gryllotalpoidea, *Teleogryllusemma* belonging to Grylloidea, *Erianthusversicolor* belonging to Eumastacoidea and *Tetrixjaponica* belonging to Tetrigoidea) were collected, with three specimens per species collected in the same region (see Table 1 for details). Before dissection, all specimens were starved for 24 hours to clear food residue from their guts and reduce chloroplast contamination. Then, all guts were dissected under sterile conditions with flame-sterilised forceps in 1X phosphate-buffered saline. The guts of each specimen were stored and frozen at -80°C before DNA extraction.

### DNA extraction and PCR amplification of the V4 region of 16S rRNA

Microbial genomic DNA was extracted from the gut samples using the phenol-chloroform method as previously described (Yang et al. 2008). Then, 0.8% agarose gel electrophoresis was performed to determine the molecular size of the extracted DNA and quantification was performed with a UV spectrophotometer. PCR amplification of the V4 region of the *16S rRNA* gene was performed using the following primers: 520F (5’-barcode+GCACCTAAYTGGGYDTAAAGNG-3’) and 802R (5’-TACNVGGGTATCTAATCC-3’). The barcode in the forward primer (520F) is a seven-base oligonucleotide sequence used to distinguish between samples in the same library. A 25-μl reaction system was used for PCR amplification, containing 0.25 μl of NEB Q5 DNA high-fidelity polymerase, 0.5 μl of dNTPs (10 mM), 5 μl of 5× PCR reaction buffer, 5 μl of 5× high GC buffer, 1 μl of DNA template, 1 μl of forward primer (10 μM), 1 μl of reverse primer (10 μM) and 11.25 μl of sterile ultrapure water. The following PCR conditions were used: initial denaturation at 98°C for 30 sec, followed by 25-27 cycles of denaturation at 98°C for 30 sec, annealing at 50°C for 30 sec and extension at 72°C for 30 sec, with a final extension step of 5 min at 72°C. PCR products were detected by performing 2% agarose gel electrophoresis and target fragments were extracted and recovered using an Axygen Axy Prep DNA Gel Extraction Kit (AXYGEN Inc., Union City, CA USA, cat#AP-GX-500). V4 amplicons were pooled and 2 × 300 paired-end sequences were analysed by Illumina MiSeq at Personal Biotech Co., Ltd. (Shanghai, China).

### Sequence analysis

To integrate raw paired-end sequences, we quality-screened for paired-end sequences in FASTQ format using Trimmomatic (v.0.36, http://www.usadellab.org/cms/index.php?page=trimmomatic) (Bolger et al. 2014). Ambiguous bases were not allowed and sequence lengths were longer than 150 bp. In addition, reads were removed if barcode errors or primer mismatches were found. We merged these reads using Flash software (v.1.2.7, http://ccb.jhu.edu/software/FLASH/) (Magoč and Salzberg 2011) and discarded unassembled reads. Chimeras were identified and removed using USEARCH (v.5.2.236, http://www.drive5.com/usearch/) in Qiime (v.1.8.0, http://qiime.org/) (Caporaso et al. 2010).

Operational taxonomic units (OTUs) were generated with sequence similarity greater than 97% using the uclust function (Edgar 2010) in Qiime. The sequence with the highest abundance for each OTU was selected as the representative sequence. Taxonomic information for each OTU was obtained by annotating the OTU representative sequence, based on the Greengenes database (Release 13.8, http://greengenes.secondgenome.com/) (DeSantis et al. 2006). A Venn diagram and the Dendrogram and Heatmap were generated using the Venn Diagram software package and ggtree software package in R. Unweighted clustering was performed using PCoA of UniFrac distance matrices.

Chao1, ACE, Shannon and Simpson indices for each sample were calculated using the summary.single command in the MOTHUR software package (http://www.mothur.org/) (Schloss et al. 2009). The relationship between the selected taxonomy group (abundant phyla and genera) and the bacterial community index (Chao1, ACE, Shannon and Simpson) was calculated using SPSS 20.0 software. Multiple-group analysis was carried out using ANOVA followed by the Tukey’s honestly significant difference test. P < 0.05 was considered as statistically significant.

## Results

### Barcoded 16S sequencing and OTUs composition

**Figure 1.**
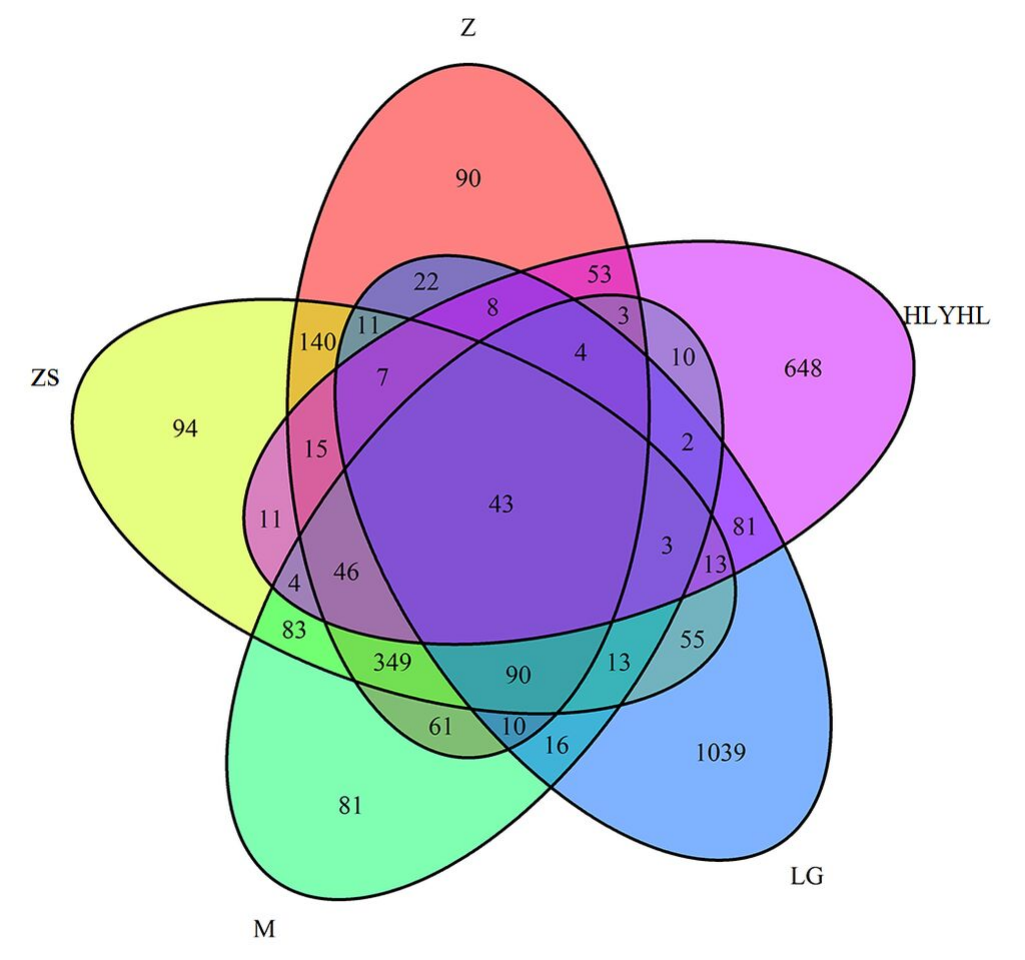
Venn diagram depicting the number of shared and exclusive bacterial OTUs in the bacterial community of five groups. Z: *Tetrixjaponica*; ZS: *Ruspolialineosa*; M: *Erianthusversicolor*; LG: *Gryllotalpaorientalis*; HLYHL: *Teleogryllusemma*.

We utilised the V4 region of the 16S rRNA amplicon to assess the gut microbiota composition of five orthopterans using Illumina MiSeq DNA metabarcode sequencing. A total of 778,780 paired-end reads were acquired from all intestinal samples, with an average read length of 450 bp. After quality control, 606,053 high-quality reads were acquired. Based on 97% species similarity and chloroplast and mitochondrial sequences and OTUs with < 0.001% abundance in all samples being removed, a total of 3,105 OTUs were obtained from all intestinal samples. The fifteen insect samples were divided into five groups, each with three samples. The number of OTUs in each group (ZS, M, HLYHL, Z and LG) was 978, 818, 951, 952 and 1,417, respectively. Amongst these, 43 OTUs present in all groups were defined as core OTUs and 94, 81, 648, 90 and 1,039 OTUs were uniquely identified in ZS, M, HLYHL, Z and LG, respectively (Fig. 1).

**Figure 2.**
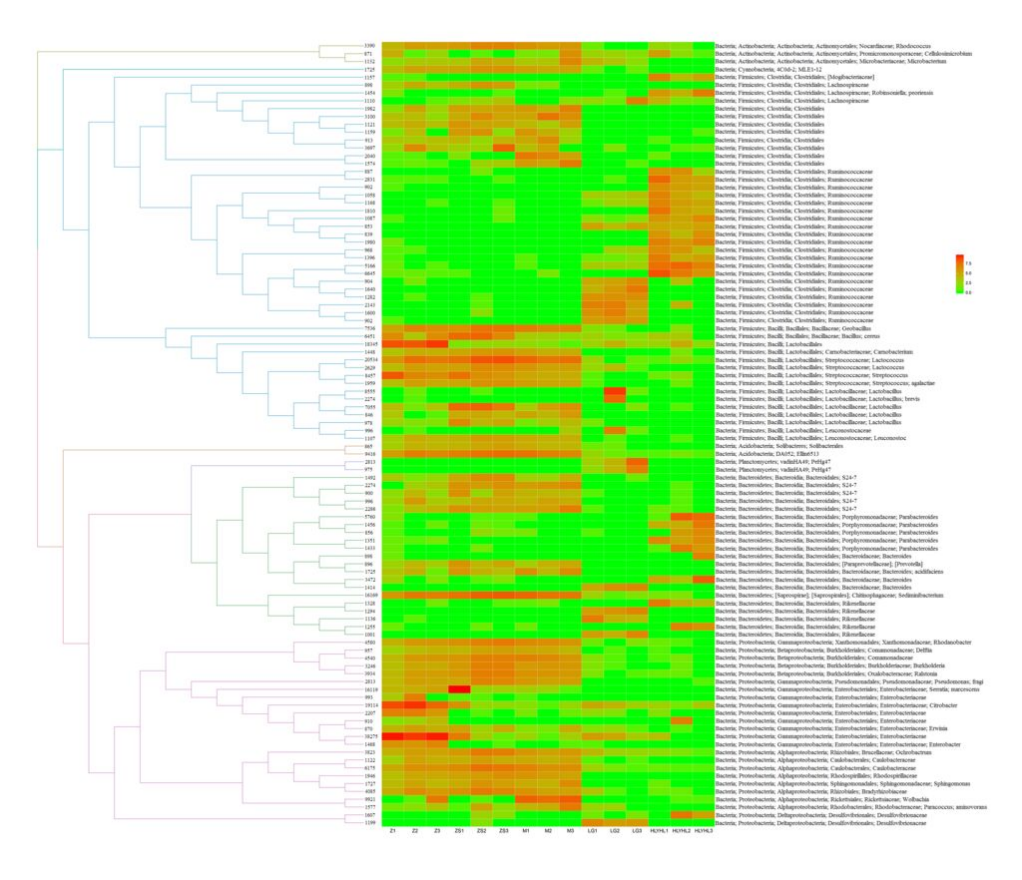
Dendrogram and heatmap of bacterial distributions of the top 100 abundant OTUs present in the microbial community of the fifteen samples. The numbers indicate the actual reads number of the OTU. The heatmap plot depicted the relative abundance of each sample and the relative values for OTUs are indicated by colour intensity. Z: *Tetrixjaponica*; ZS: *Ruspolialineosa*; M: *Erianthusversicolor*; LG: *Gryllotalpaorientalis*; HLYHL: *Teleogryllusemma*.

**Figure 3.**
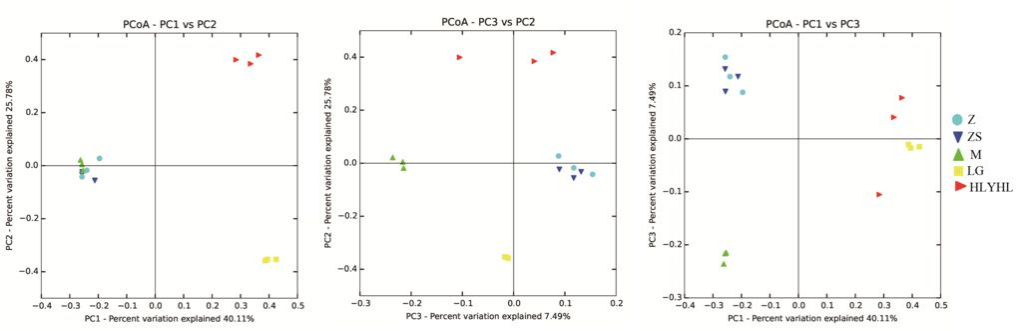
PCoA plot based on an unweighted UniFrac distance matrix depicting differences in the composition of the gut microbiota of the five groups. In the unweighted UniFrac analysis of the gut samples, the first principal coordinate, explained 40.11% of sample variation and separated groups of LG and HLYHL from others. The third principal coordinate (7.49% of sample variation) separated groups (M) from others. Z: *Tetrixjaponica*; ZS: *Ruspolialineosa*; M: *Erianthusversicolor*; LG: *Gryllotalpaorientalis*; HLYHL: *Teleogryllusemma*.

The Dendrogram and heatmap revealed the differences of the top 100 OTUs amongst the 15 samples (Fig. 2). The most abundant and prevalent OTUs belonged to the families Ruminococcaceae (belonging to Firmicutes) and Enterobacteriaceae (belonging to bacteria). Ruminococcaceae was very abundant across the samples of LG and HLYHL, but virtually absent from Z, ZS and M. On the contrary, Enterobacteriaceae was very abundant across Z, but ZS, M, LG and HLYHL were relatively absence (Fig. 2). From the 100 most prevalent OTUs, 47 belonged to Firmicutes, 24 belonged to bacteria, 21 belonged to Bacteroidetes and there were a few Acidobacteria, Actinobacteria, Cyanobacteria and Planctomycetes. Within the Firmicutes, all OTUs belonged to Clostridiales and Lactobacillales order, except for two Bacillales OTUs. Within the Bacteroidetes, all OTUs, except for one [Saprospirales] OTU, belonged to Bacteroidales order (Fig. 2). The Principal Coordinates Analysis (PCoA), based on an unweighted UniFrac distance matrix, revealed differences in microbiota composition for different groups; the bacterial composition of each group were distinctly different, except for Z and ZS (Fig. 3). The ANOSIM and Adonis analysis (P = 0.001 and P = 0.001, respectively) also indicated different groups differed significantly.

### Analysis of alpha diversity

Gut microbiota alpha diversity was estimated using alpha diversity curves (rarefaction curves and Shannon–Wiener curves) and alpha diversity indices (Chao1, ACE, Simpson and Shannon indices). The rarefaction curves (Amato et al. 2013) and Shannon–Wiener curves (Wang et al. 2012) for each sample are shown in Suppl. material 1: Figure S1. The rarefaction curves reached a saturation phase at 20,000 reads, indicating sufficient recovery of the OTUs present in the datasets. The Shannon-Wiener curves also reached saturation, indicating the addition of more sequences did not alter the saturation of microbial diversity.

The diversity indices for each sample are shown in Table 2. The Chao1 and ACE indices reflected microbial community richness and the Simpson and Shannon indices reflected microbial community diversity. ANOVA indicated significant differences for Chao1 (P = 0.001), ACE (P = 0.002) and Shannon (P = 0.027) and Simpson (P = 0.100) showed no difference (Table 2). According to the Chao1 and ACE indices, the bacterial richness of LG was significantly higher than ZS, HLYHL, Z and M (P < 0.05) (Suppl. material 1: Figure S2A). According to the Shannon Index, the bacterial diversity of LG was significantly higher than Z (P < 0.05), but the Simpson Index showed no difference amongst the five groups (Suppl. material 1: Figure S2B, C).

### Microbial composition and intestinal sample abundance

Amongst the identified sequences, a total of 219, 133, 69, 48 and 24 microbes at the genus, family, order, class and phylum taxonomic levels, respectively, were identified across all samples. Table 3 shows the gut microbial composition at different taxonomic levels. In this study, we primarily compared and analysed microbial composition and abundance at the genus and phylum taxonomic levels.

**Figure 4.**
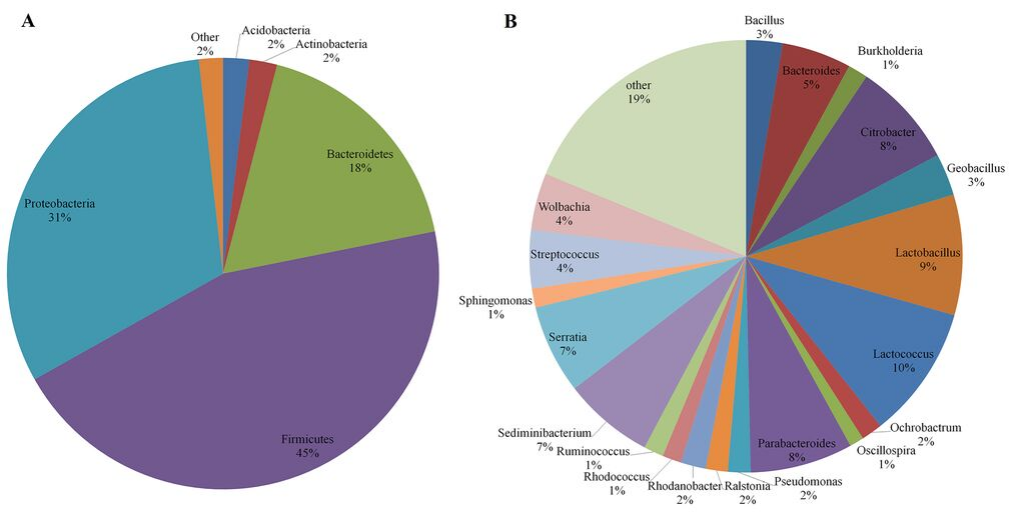
Distribution of the gut microbiota composition. **A** Five groups at phylum level; **B** Five groups at genus level.

Amongst 24 phyla, Firmicutes (45.0%), bacteria (31.4%), Bacteroidetes (17.8%), Actinobacteria (2.1%) and Acidobacteria (2.0%) were present in all samples and abundant in the majority of samples, representing more than 98% of total sequences (Fig. 4A). The bacterial composition and abundance of distinct phyla differed amongst the five groups (Suppl. material 1: Figure S3A). Firmicutes was the most predominant phylum in ZS, LG and HLYHL, accounting for 42.0%, 57.6% and 62.2% of sequences, respectively. bacteria was the most predominant phylum in Z and M, accounting for 59.6% and 36.9% of sequences, respectively. Composition and abundance at the phylum taxonomic level were investigated for each gut microbiota sample (Suppl. material 1: Figure S3B). For Z1, Z2 and Z3, bacteria was the most predominant phylum, accounting for 63.4%, 60.0% and 55.5% of sequences, respectively. For LG1, LG2 and LG3, Firmicutes was the most predominant phylum, accounting for 43.4%, 76.0% and 49.0% of sequences, respectively. For HLYHL1 and HLYHL2, Firmicutes was the most predominant phylum, accounting for 85.9% and 51.6% of sequences, respectively; however, Bacteroidetes was the most predominant phylum for HLYHL3, accounting for 48.8% of sequences. In M1, M2, and ZS2, ZS3, the most predominant phylum was Firmicutes (accounting for 38.5%, 43.9%, 49.3% and 44.4% of sequences, respectively); however, bacteria predominated in M3 and ZS1 (accounting for 41.6% and 49.9% of sequences, respectively).

Amongst 219 genera, *Lactococcus* (9.95%), *Lactobacillus* (9.00%), *Citrobacter* (7.87%), *Parabacteroides* (7.67%), *Sediminibacterium* (6.77%), *Serratia* (6.65%), *Bacteroides* (5.18%), *Streptococcus* (4.37%), *Wolbachia* (4.27%), *Geobacillus* (3.14%), *Bacillus* (2.72%), *Rhodanobacter* (1.89%), *Pseudomonas* (1.69%), *Ralstonia* (1.63%), *Ochrobactrum* (1.58%), *Burkholderia* (1.49%), *Ruminococcus* (1.48%), *Sphingomonas* (1.42%), *Rhodococcus* (1.41%) and *Oscillospira* (1.07%) were the most abundant genera, accounting for more than 81% of total sequences (Fig. 4B). Amongst these, *Bacteroides*, *Parabacteroides*, *Bacillus*, *Lactococcus*, *Oscillospira*, *Ruminococcus*, *Ochrobactrum* and *Citrobacter* were present in all samples. Microbial composition and abundance varied significantly across groups (Suppl. material 1: Figure S4A). *Citrobacter* was the most predominant genus in Z (accounting for 39.8% of sequences), but its abundance was very low in ZS, M, LG and HLYHL. *Serratia* was the most predominant genus in ZS (accounting for 18.3% of sequences), but was not found in LG and HLYHL. *Wolbachia*, *Lactobacillus* and *Parabacteroides* were the most predominant genera in M (accounting for 17.1% of sequences), LG (accounting for 50.2% of sequences) and HLYHL (accounting for 49.0% of sequences), respectively. Microbial composition and abundance in different samples within the same groups also varied significantly (Suppl. material 1: Figure S4B). *Serratia* was the most predominant genus in ZS1, but *Lactococcus* was the most predominant genus in ZS2 and ZS3. *Lactobacillus* was the most predominant genus in LG2, but demonstrated very low abundance in LG1 and LG3.

### Analysis of differences amongst groups

**Figure 5.**
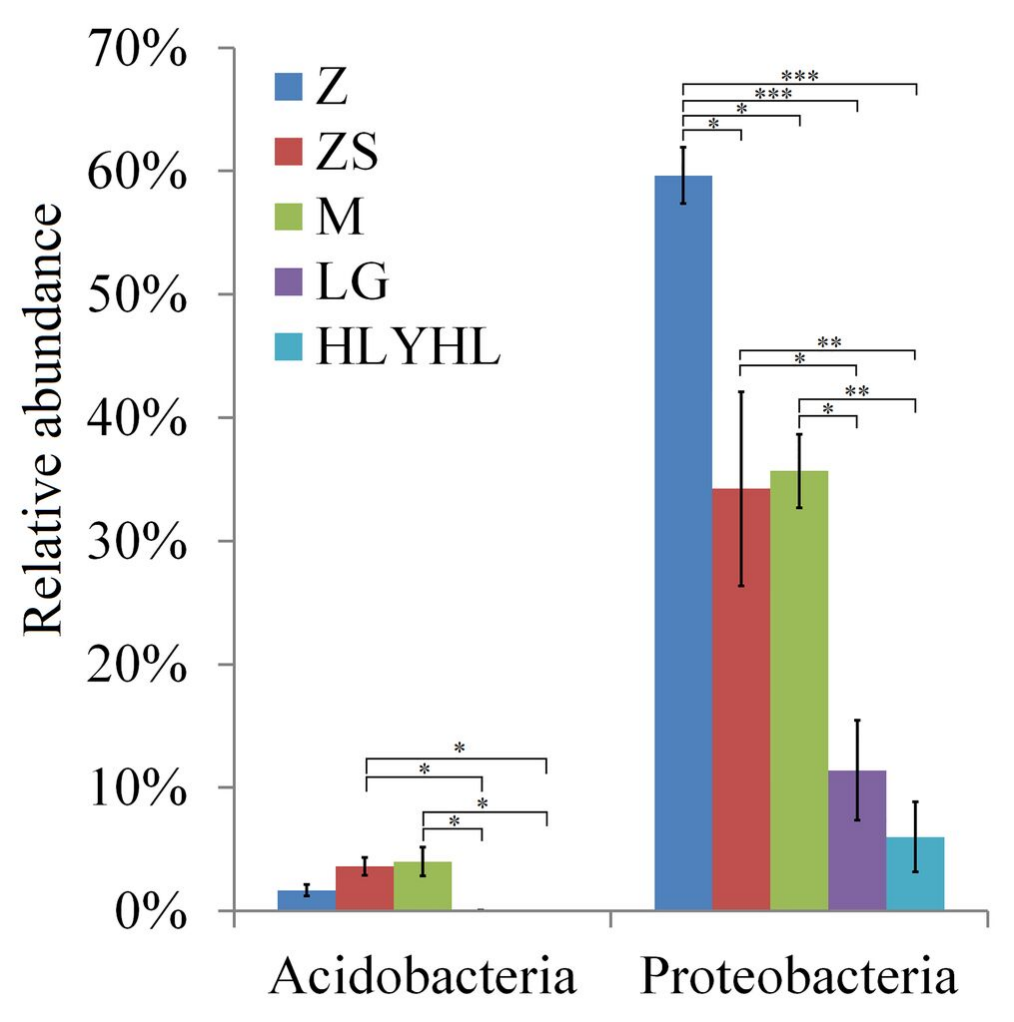
The relative abundance (% of individual taxonomic group) of Acidobacteria and bacteria present in the microbial community of the different groups. Differences were analysed by employing ANOVA analysis and Tukey Post Hoc HSD Significance Test (* P < 0.05, ** P < 0.01, *** P < 0.001). Z: *Tetrixjaponica*; ZS: *Ruspolialineosa*; M: *Erianthusversicolor*; LG: *Gryllotalpaorientalis*; HLYHL: *Teleogryllusemma*.

At the phylum level, we analysed the differences in Firmicutes, bacteria, Bacteroidetes, Actinobacteria and Acidobacteria in different groups. Amongst these, Acidobacteria (P < 0.01) and bacteria (P < 0.001) demonstrated significant differences and Actinobacteria, Bacteroidetes and Firmicutes showed no differences. We further calculated multiple comparisons to show differences between each two groups of Acidobacteria and bacteria, the relative abundance of the phylum bacteria in Z was mostly significantly higher than others and the relative abundance of the phylum Acidobacteria in ZS and M were significantly higher than LG and HLYHL (Fig. 5).

Amongst the 20 most abundant genera, ANOVA indicated significant differences for *Lactococcus* (P < 0.05), *Citrobacter* (P < 0.001), *Parabacteroides* (P < 0.01), *Sediminibacterium* (P < 0.01), *Wolbachia* (P < 0.001), *Geobacillus* (P < 0.01), *Bacillus* (P < 0.05), Rhodanobacter (P < 0.05), *Pseudomonas* (P < 0.05), *Ralstonia* (P < 0.01), *Ochrobactrum* (P < 0.05), *Burkholderia* (P < 0.01) and *Rhodococcus* (P < 0.01) (Suppl. material 1: Figure S5).

## Discussion

Based on the results obtained for 15 samples across five orthopteran species using DNA metabarcoding, the predominant phyla in the insect gut were Firmicutes and bacteria, representing 70.1% of total sequences. This result is quite similar to those obtained in previous studies. Yun et al. studied gut samples from 305 individuals belonging to 218 species in 21 taxonomic orders and found the predominant phyla to be Firmicutes and bacteria, representing 82.8% of total sequences (Yun et al. 2014). Additionally, Colman et al. studied 62 insect gut samples and found Firmicutes and bacteria to be the predominant phyla, comprising 79.1% of total sequences (Colman et al. 2012). Bacteroidetes, the third most predominant phylum, generally produces butyrate, a chemical thought to have antineoplastic properties, in the mammalian gut (Kim and Milner 2007).

According to our study, the predominant genera in the gut were *Lactococcus* and *Lactobacillus*, belonging to the order Lactobacillales and the class Bacilli. Bacilli species reportedly exert beneficial effects in terms of preventing intestinal disorders and reducing inflammation (Hong et al. 2005); they are also the microbial communities responsible for biogas production (Schlüter et al. 2008). Lactobacillales are known for their beneficial effects in insects, such as their ability to mediate insecticide resistance (Xia et al. 2013), modulate the microflora composition to protect the host against infections (Ouwehand et al. 2002), promote intestinal peptidase expression, increase intestinal proteolytic activity (Erkosar et al. 2015) and enhance the systemic production of host ecdysone and insulin-like peptides (Storelli et al. 2011). As described in several reports, *Wolbachia* induce male-killing, regulate host reproduction (Jiggins et al. 2000, Hiroki et al. 2002, Sebastien et al. 2012) and defend some insects against natural enemies (Hedges et al. 2008, Teixeira et al. 2008). *Wolbachia* (14.1%) was the most prevalent genera in a study of 305 individuals belonging to 21 taxonomic orders (Yun et al. 2014). However, in our study, *Wolbachia* abundance only reached 4.27% and ANOVA results indicated *Wolbachia* differed significantly amongst the five groups. The abundance of *Wolbachia* was highest in M and there were no *Wolbachia* bacteria in ZS, LG and HLYHL. Jeyaprakash and Hoy (2000) and Russell et al. (2012) observed *Wolbachia* strains in Orthoptera and Yun et al. showed *Wolbachia* to be the dominant species in Orthoptera (Yun et al. 2014). We compared *Wolbachia* in five species of Orthoptera and found this genus in *E.versicolor* and *T.japonica*, but not *R.lineosa*, *G.orientalis* or *T.emma*.

When comparing gut bacteria amongst samples, we identified differences in diversity and abundance. Stanley et al. analysed samples from 207 chicken caecal microbiota across three similar trials and demonstrated the ability of host genes and environmental factors to alter the composition of the intestinal microflora (Stanley et al. 2013). A previous study investigating Mormon crickets suggested gut bacteria are either acquired from the environment in each generation or are not restricted over appreciable periods of evolutionary time (Smith et al. 2017). Dynamic variations in the gut microbiota are attributable to ecological conditions in the gut, including pH levels, redox conditions, oxygen levels and biologically active compounds (Dillon and Dillon 2004, Engel and Moran 2013). Variations are also attributable to ecological relationships between gut microorganisms. Positive interactions may promote the symbiosis of intestinal microbiota, while negative interactions inhibit symbiosis, resulting in changes in the gut microflora composition amongst individual hosts (Coyne et al. 2005, Donohoe et al. 2011, Rosenthal et al. 2011).

To evaluate the relationships between the gut microbiota and host in five species, we collected 15 samples and classified them into five groups. Amongst the six most abundant phyla, ANOVA analysis revealed that Acidobacteria and bacteria differed significantly. bacteria abundance was highest in Z, followed by ZS, M, LG and HLYHL. Acidobacteria abundance was highest in ZS, followed by M and Z and low abundance in LG and HLYHL. Bacteroidetes, Cyanobacteria and Firmicutes did not differ significantly. Amongst the 20 most abundant genera, 13 to 20 were significantly different. Of these, all were low in LG and HLYHL with the exception of *Parabacteroides*. According to our PCoA and heatmap analysis, different individuals in the same group had relatively close relationships and, thus, bacterial community composition similarity was higher in same-group individuals than in different-group individuals. Alpha diversity analysis showed significant differences for Chao1, ACE and Shannon, illustrating higher bacterial community richness and diversity in the different groups.

In summary, our study revealed the composition and diversity of the gut microbiota of 15 individuals belonging to five orthopteran species using DNA metabarcode sequencing. The results revealed a bacterial community composition comprising 24 phyla and 219 genera. The most abundant phyla were Firmicutes and bacteria and the most abundant genera were Lactococcus and Lactobacillus. We also compared differences in bacterial composition of distinct species at the phylum and genus levels. The results suggested the gut bacteria composition differed significantly across the five species.

## Data resources

The raw data are available at the National Center for Biotechnology Information (NCBI) SRA (https://www.ncbi.nlm.nih.gov/sra/): SRR20722952 - SRR20722966.

## Disclosure

The authors declare that the research was conducted in the absence of any commercial or financial relationships that could be construed as a potential conflict of interest.

## Supplementary Material

06521778-B7D1-5348-9D50-92DB6E039CC8 10.3897/BDJ.11.e98162.suppl1 Supplementary material 1 The gut microbiota diversity of five Orthoptera insects determined by DNA metabarcoding Data type images File: oo_767535.docx https://binary.pensoft.net/file/767535 Yantong Liu1, Lina Zhao2, Zhongying Qiu1, Hao Yuan1*

## Figures and Tables

**Table 1.** Information of studied samples.

**Superfamily**	**Species**	**SampleID**		**Location**	**Date**
Tetrigoidea	*Tetrixjaponica*	Z	Z1	Shaanxi, Xi’an	21/08/2016
			Z2	Shaanxi, Xi’an	21/08/2016
			Z3	Shaanxi, Xi’an	21/08/2016
Tettigoniidae	*Ruspolialineosa*	ZS	ZS1	Shaanxi, Xi’an	22/08/2016
			ZS2	Shaanxi, Xi’an	22/08/2016
			ZS3	Shaanxi, Xi’an	22/08/2016
Eumastacoidea	*Erianthusversicolor*	M	M1	Guangdong, Ruyuan	15/09/2016
			M2	Guangdong, Ruyuan	15/09/2016
			M3	Guangdong, Ruyuan	15/09/2016
Gryllotalpidae	*Gryllotalpaorientalis*	LG	LG1	Henan, Nanyang	29/08/2016
			LG2	Henan, Nanyang	29/08/2016
			LG3	Henan, Nanyang	29/08/2016
Gryllidae	*Teleogryllusemma*	HLYHL	HLYHL1	Shaanxi, Xi’an	21/08/2016
			HLYHL2	Shaanxi, Xi’an	21/08/2016
			HLYHL3	Shaanxi, Xi’an	21/08/2016

**Table 2.** Diversity index of each sample.

**SampleID**	**Chao1**	**ACE**	**Simpson**	**Shannon**
Z1	371	478.18	0.78	3.36
Z2	522	648.34	0.89	4.82
Z3	446	577.26	0.83	4.11
ZS1	498	582.61	0.86	5.11
ZS2	665	788.12	0.97	6.43
ZS3	594	694.67	0.97	6.43
M1	339	395.71	0.89	4.83
M2	306	396.69	0.77	3.56
M3	579	629.24	0.98	7.19
LG1	865	865.00	0.99	7.87
LG2	898	969.45	0.92	6.36
LG3	932	971.13	0.98	7.62
HLYHL1	436	468.90	0.96	6.18
HLYHL2	582	621.68	0.97	6.76
HLYHL3	602	621.76	0.98	6.87
p-value	0.001	0.002	0.100	0.027

**Table 3.** The gut microbial composition at different taxonomic levels.

**SampleID**	**Phylum**	**Class**	**Order**	**Family**	**Genus**	**OTUs**
Z1	13	25	37	84	111	581
Z2	18	33	41	93	131	694
Z3	16	29	41	92	125	616
ZS1	15	26	36	83	114	624
ZS2	16	28	42	95	136	811
ZS3	16	31	43	91	124	726
M1	15	29	40	90	113	515
M2	12	25	35	83	105	455
M3	16	31	38	95	151	656
LG1	16	28	39	63	83	1049
LG2	14	27	39	66	78	1104
LG3	15	25	35	55	65	1080
HLYHL1	5	13	20	40	41	512
HLYHL2	7	15	25	42	50	725
HLYHL3	7	14	24	34	36	680
Z	18	35	48	105	160	955
ZS	18	36	53	109	155	980
M	17	35	46	107	165	827
LG	18	34	45	75	101	1417
HLYHL	8	18	29	51	65	951
Total	24	48	69	133	219	3105
