# Supplementary material for: The gut microbiota diversity of five Orthoptera (Insecta, Polyneoptera) insects determined by DNA metabarcoding
Source: Biodivers Data J. 2023 Mar 15;11:e98162. doi: 10.3897/BDJ.11.e98162 (PMC10848783; doi:10.3897/BDJ.11.e98162)
Supplement: Supplementary material 1 — The gut microbiota diversity of five Orthoptera insects determined by DNA metabarcoding [file bdj-11-e98162-s001.docx]

The gut microbiota diversity of five Orthoptera insects determined by DNA metabarcoding

Yantong Liu^1^, Lina Zhao^2^, Zhongying Qiu^1^, Hao Yuan^1*^

^1^ School of Basic Medical Sciences, Xi’an Medical University, Xi’an, China; yantong_l@163.com (Y.L.); qiuzhongying11@126.com (Z.Q.)

^2^ College of Life Sciences, Shaanxi Normal University, Xi’an, China; Linazhao@snnu.edu.cn (L.Z.)

*** Correspondence:**Hao Yuan
2022020001@xiyi.edu.cn

**Supplementary** **Figure**


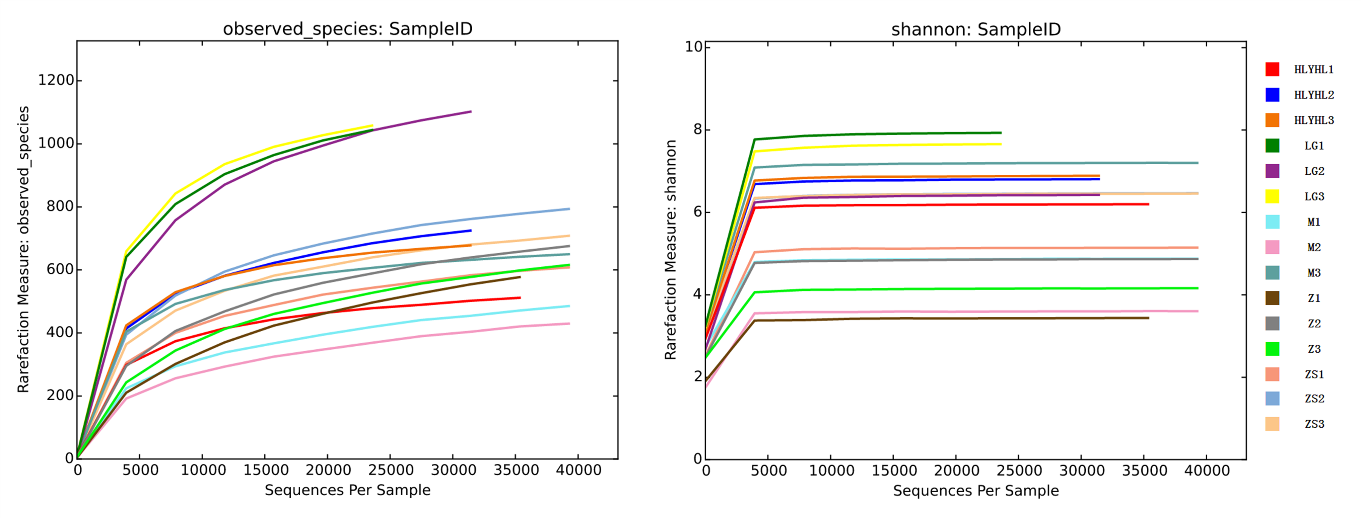


**Figure S1. The rarefaction curves and Shannon–Wiener curves of 15 samples.**

(A) The rarefaction curves of 15 samples. (B) The Shannon–Wiener curves of 15 samples.


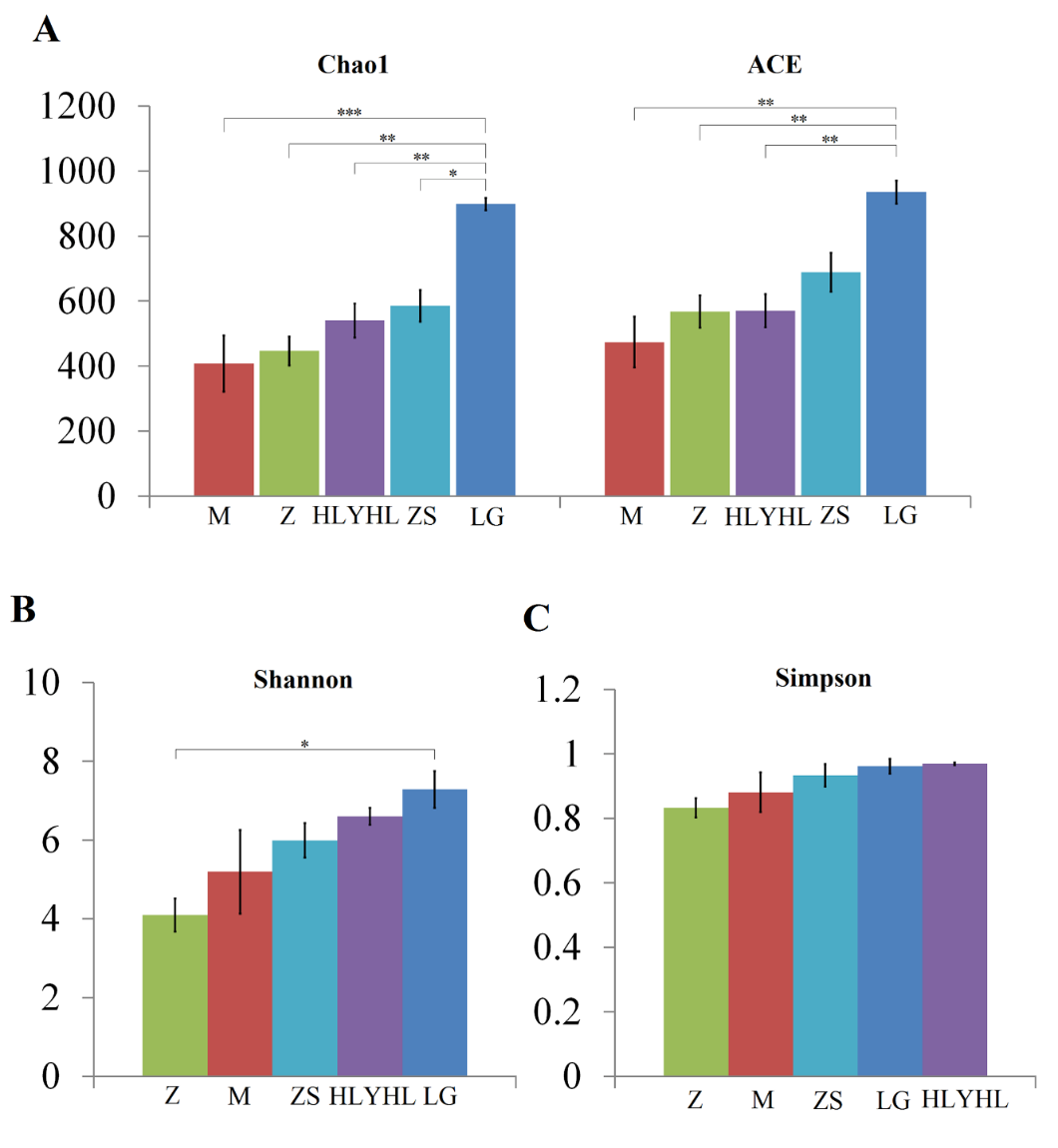


**Figure S2. Diversity indices of the bacterial communities of the five groups.**

(A) Differences of Chao1 and ACE indices. (B) Differences of Shannon index. (C) Differences of Simpson index. Differences of diversity indices were analyzed by employing ANOVA analysis and Tukey Post Hoc HSD Significance Test (* P <0.05, ** P <0.01, *** P <0.001).


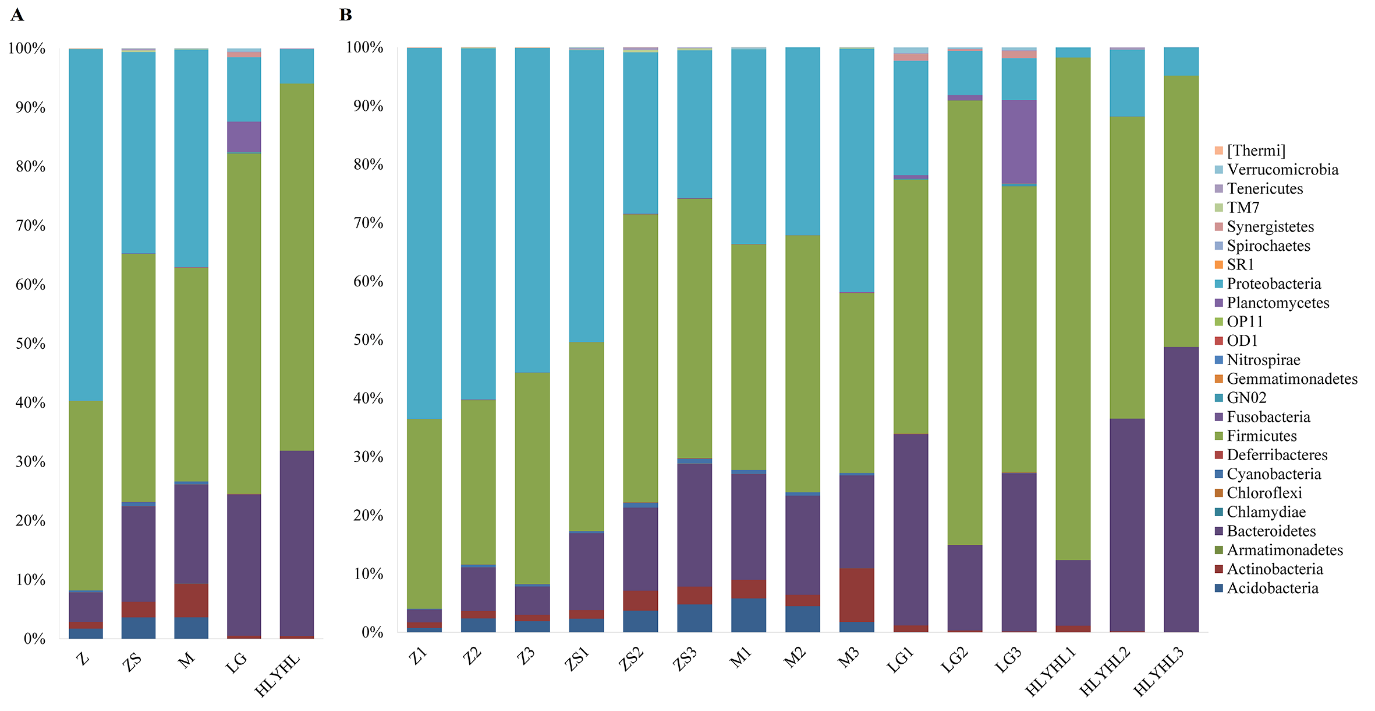


**Figure S3. Phyla distribution of gut flora.**

(A) Phyla distribution of gut flora of five groups. (B) Phyla distribution of gut flora of 15 samples.


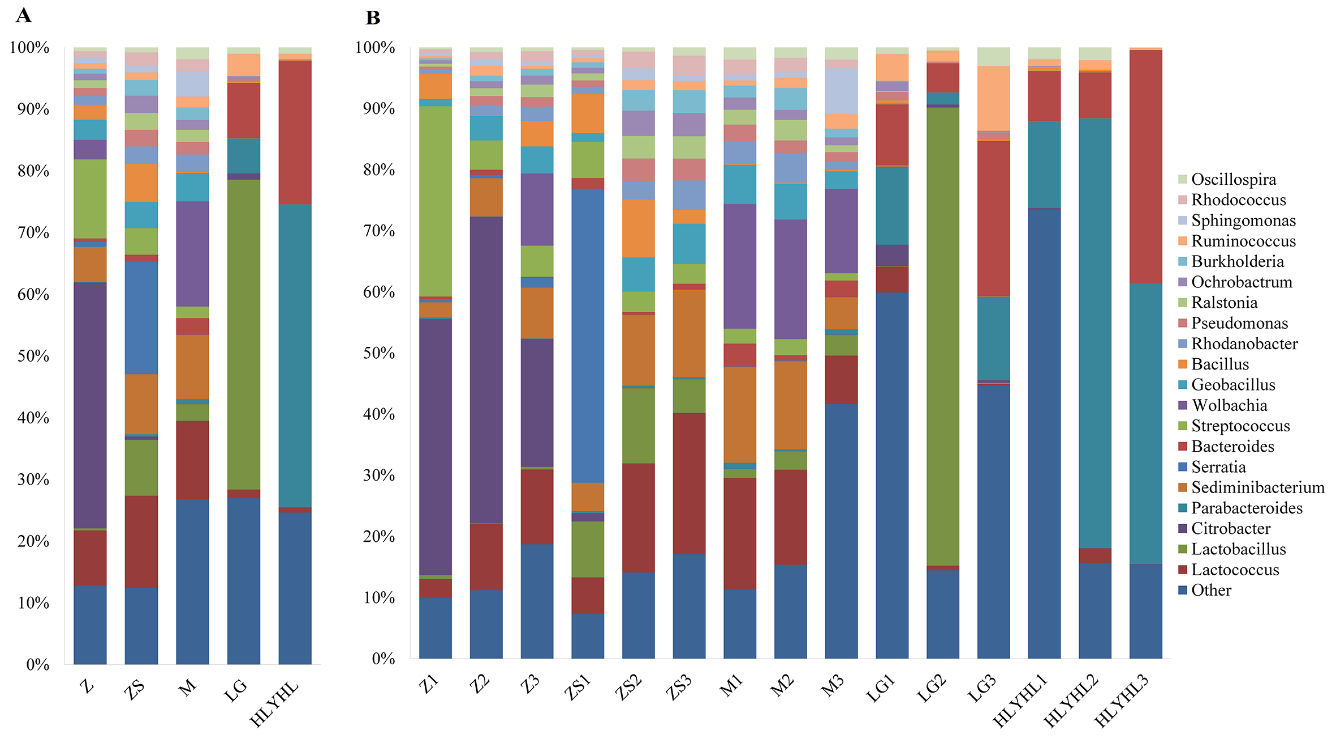


**Figure S4. Genus distribution of gut flora.**

(A) Genus distribution of gut flora of five groups. (B) Genus distribution of gut flora of 15 samples. The genera with relative abundance larger than 1.0% were listed in the figure, respectively. The genera with relative abundance lower than 1.0% in all groups were copolymerized in “Other”, respectively.


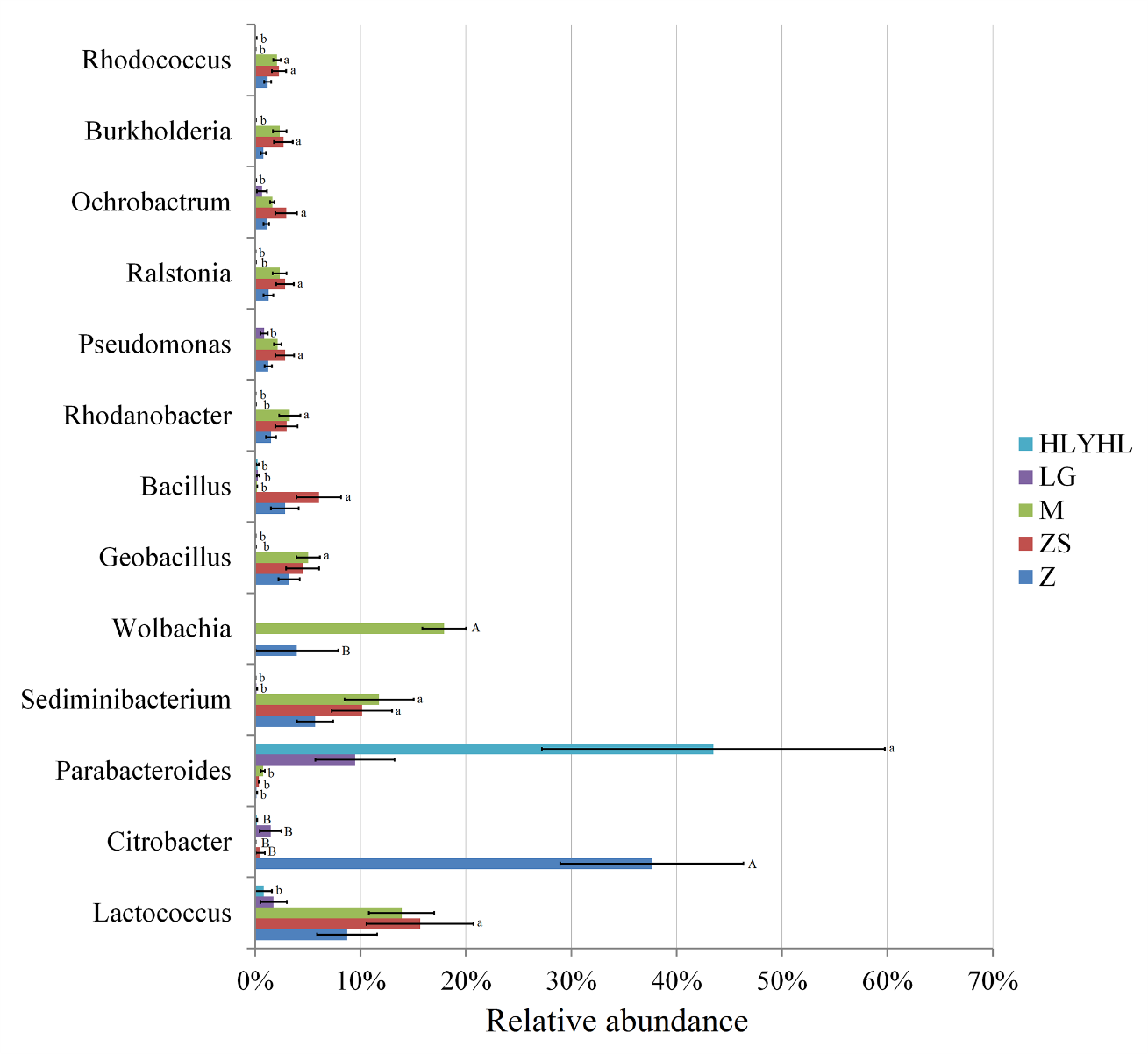


**Figure S5. The relative abundances of 13 distinct genera in five groups were significantly different.**

Bars with different letters represent significant differences among the five different groups following Tukey Post Hoc HSD Significance Test. ^a,b^ The correlation is significant at a level of 0.05; ^A,B^ the correlation is significant at a level of 0.01.
